# Researching New Methods of Screening for Adverse Pregnancy Outcome: Lessons from Pre-eclampsia

**DOI:** 10.1371/journal.pmed.1001274

**Published:** 2012-07-31

**Authors:** Gordon C. S. Smith

**Affiliations:** Department of Obstetrics and Gynaecology, Cambridge University, Cambridge, United Kingdom

## Abstract

Gordon Smith argues for more and better research in screening for pregnancy outcomes, using the example of previous trials in pre-eclampsia.

**Summary Points**

- Screening low-risk women for the risk of adverse pregnancy outcome, such as pre-eclampsia, is still largely based on clinical assessment, due to negative trials of new methods.
- I argue that previous studies have weaknesses in their design and have focused on preterm pre-eclampsia despite the lack of clearly effective interventions to improve outcome.
- A significant proportion of severe pre-eclampsia occurs at term and could plausibly be prevented by novel screening tests and early term delivery of high-risk women.
- Novel screening programmes focused on preventing adverse pregnancy outcome at term are also more likely to be translatable into low-income settings, where the majority of maternal and perinatal deaths occur.

## Screening for Adverse Pregnancy Outcome

Complications of pregnancy contribute to a substantial proportion of the global burden of disease [1]. Most adverse pregnancy outcomes occur to women who lack obvious risk factors. However, despite many years of research, the current approach to screening low-risk women for complications such as pre-eclampsia and stillbirth is still based around serial measurement of blood pressure, urinalysis, and symphysis-fundal height [2]. In this Essay, I argue that the failure to develop more effective programmes of screening and intervention is due in part to limitations in the approach to research in this area. The arguments will be illustrated using utero-placental Doppler flow velocimetry and screening for pre-eclampsia as an example. Many of the principles are relevant for trials of other methods of screening and intervention to prevent pre-eclampsia, for other adverse pregnancy outcomes (stillbirth in particular), and in other areas of medicine.

## Existing Trials of Screening for Pre-eclampsia

Research on the biological pathways that lead to pre-eclampsia focus on the placenta. The current view is that impaired invasion of the maternal uterine resistance vessels by the invading extravillous trophoblast is a key determinant. Trophoblast invasion can be assessed clinically by Doppler flow velocimetry of the uterine arteries [3]. A high resistance pattern of flow at 23 weeks gestational age is a sensitive and specific predictor of early onset pre-eclampsia in an unselected population [3]. However, meta-analysis of randomised controlled trials indicates that routine use of utero-placental Doppler in low-risk women had no effect on pregnancy outcome [4].

## Existing Interventions to Reduce the Risk of Pre-eclampsia

A successful screening programme consists of two components. The first is that women at high risk of a disease, or in the early phase of a disease, are identified. The second is that these women are then treated with an intervention that results in a better outcome than if treatment had been initiated when the disease became clinically apparent. A screening programme could, therefore, yield a negative result due either to failure of the screening test or failure of the intervention. A detailed analysis of the literature conducted for the NICE Guideline on Hypertension in Pregnancy demonstrated that, of the many candidate interventions evaluated (dietary, lifestyle, nutritional, and pharmacological), only low dose aspirin had been clearly shown to reduce the risk of pre-eclampsia in high-risk women [5]. However, aspirin only reduced the risk of pre-eclampsia by about 17% [6]. Hence, there are currently no highly effective disease modifying therapies available for women who screen as high risk for pre-eclampsia.

## Designing Trials

**Figure 1:**
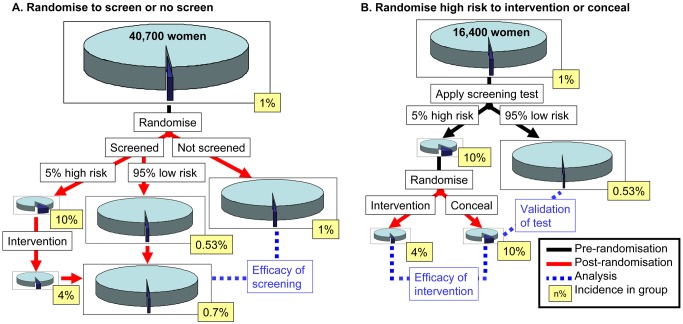
The effect of study design on sample size calculations and conclusions that can be drawn from screening studies. (A) Women are randomised to having or not having the screening test performed. (B) Women have the screening test performed and those who screen as high risk are randomised to having an intervention or having the result concealed. The number of women required (indicated in the top of each figure) is from a sample size calculation for 90% power to detect an effect at *p*<0.05 (two sided). In panel (A), it is the number of women who need to be consented to be randomised to being screened or not screened and the calculation is based on the rate of the primary outcome comparing screened versus not-screened. In panel (B), it is the number of women who need to be screened and it is based on the rate of the primary outcome comparing intervention versus no intervention among women who screened positive. Both calculations assume an outcome with a background incidence of 1%, a screen positive rate of 5%, and a positive predictive value of 10% (hence a positive likelihood ratio of 11). Based on these parameters, the 95% of the population who screened negative would have a 0.53% incidence, giving a negative predictive value of 99.5% and a negative likelihood ratio of 0.52. The intervention is assumed to reduce the risk of the outcome by 60%. See Table S1 for power calculations for both designs using other values of screening performance or intervention efficacy.

The simplest way to evaluate a screening programme is to randomise women to be screened or not screened. However, an alternative approach is to screen all women and to randomise those who screen positive to the intervention or to having the result concealed (see Figure 1). The second approach has two advantages. First, it requires a smaller sample size (Table S1). In the example illustrated by Figure 1, it reduced the required number of participants by 60%. Second, if a trial of randomisation to the screening test yields a negative result, it is impossible to assess whether it is because of failure of the screening test or failure of the intervention. In contrast, if high-risk women are randomised to an intervention or to having the result concealed, it allows the effectiveness of the screening test and the intervention to be evaluated separately in the same study.

## Critical Assessment of Trials of Screening Using Utero-Placental Doppler

Reviewing the trials included in the meta-analysis of screening low-risk women using utero-placental Doppler indicates that all of them randomised women to being screened or not being screened. The power calculation in Figure 1 indicates that, where the background incidence of the outcome is 1% (as is the case for severe pre-eclampsia), this study design would require recruiting >40,000 women, even where the test is highly informative and the intervention is highly effective. The meta-analysis of routine Doppler contains 14,185 individuals [4]. Furthermore, none of the trials included in the meta-analysis had a specific intervention other than revealing the result, with or without a programme of increased surveillance.

## Designing Interventions for Future Trials

A key intervention in the management of women with established pre-eclampsia is to deliver the infant, but the effect depends on the gestational age. Routine delivery preterm increases neonatal morbidity without improving maternal outcome [7]. However, even among women with mild pre-eclampsia, routine delivery at 37 weeks gestational age improved outcome [8]. It is plausible that delivery at 37 weeks gestational age would also be an effective intervention for women who were asymptomatic but were at high risk of severe pre-eclampsia at term. When considering the most severe consequences of pre-eclampsia, there tends to be a focus on disease resulting in preterm birth. However, a significant proportion of severe disease occurs at term. For example, an analysis of data from Canada demonstrated that two-thirds of cases of eclamptic seizures occurred at term [9]. Furthermore, a review of cases of severe pre-eclampsia in 16 maternity units in Yorkshire (United Kingdom) demonstrated that one-third of neonatal deaths were of infants born at term [10].

## Designing Screening Tests for Complications at Term

**Figure 2:**
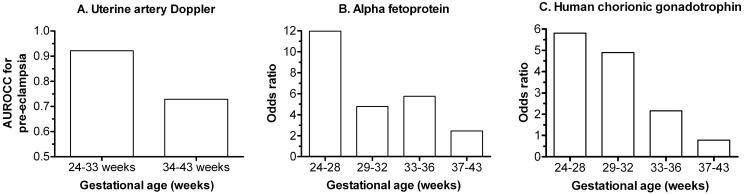
Timing of screening tests and the subsequent risk of complications, in relation to gestational age at delivery. (A) Area under the receiver operating characteristic curve (AUROCC) for pre-eclampsia at different gestational ages in relation to uterine artery Doppler flow velocimetry performed at around 23 weeks gestational age. The origin of the *y*-axis is set to 0.5 as this is the value where the test is non-informative (data from [3]). (B) Adjusted odds ratio for stillbirth at different gestational ages among women with maternal serum alpha fetoprotein levels in top 1% at 15–20 weeks. (C) As (B) except top 1% of maternal serum levels of human chorionic gonadotrophin (data from [13]).

A significant body of work demonstrates that many complications of late pregnancy may be determined in the first trimester [11]. While these analyses provide interesting insights into possible biological determinants of adverse outcome, clinical studies of both biochemical and ultrasonic screening tests for placental dysfunction indicate that both are more strongly associated with complications temporally closer to the time of measurement (Figure 2). Hence, screening for adverse outcome at term may be most informative if it includes an assessment around 34–36 weeks gestation. This could be combined with earlier data and clinical details to provide an individualised risk of pre-eclampsia at term. However, some current high quality prospective cohort studies, such as nuMoM2b (http://www.clinicaltrials.gov/ct2/show/NCT01322529) and SCOPE (http://www.scopestudy.net/) only assess screening tests up to 29 and 24 weeks gestational age, respectively.

## Screening and Intervention to Prevent Severe Pre-eclampsia

On the basis of the above, I propose that the current lack of progress in reducing the burden of morbidity and mortality in relation to pre-eclampsia (as well as other serious adverse outcomes of pregnancy, such as stillbirth) may be best addressed by the following approach. 1. High quality non-interventional studies should characterise the screening performance of existing and novel tests in low-risk populations. 2. The gestational age dependence of risk prediction should be assessed. 3. Measurements should be made in all three trimesters, including a late preterm assessment (34–36 weeks). 4. Initial efforts should focus on identifying women at high risk of developing severe disease at term. 5. Screening algorithms for severe disease at term should be evaluated in trials where all women are screened and high-risk women are randomly allocated to having the result revealed with a recommendation for delivery at 37 weeks, or having the result concealed. Such trials would allow proof of principle of both the screening test and the intervention, prior to larger and more expensive trials of screening versus no screening.

## Improving Outcomes in Low-Income Settings

Globally, approximately 350,000 women die during or shortly after pregnancy, 2.6 million babies are stillborn, and 3.2 million liveborn children die in the first month of life [1],[12]. Although most research on improved methods of screening and prevention of maternal and perinatal mortality occurs in high-income settings, most deaths occur in low- and middle-income settings. Focusing on screening for disease at preterm gestations is less likely to be translatable to low- and middle-income settings, as these countries often have limited provision of neonatal intensive care. However, if novel biomarkers are developed to identify women at high risk of severe term pre-eclampsia, screening women in late pregnancy in low- and middle-income settings could be feasible. For example, community testing using urine or capillary blood could be used to identify women who should be transferred to a health care facility for early term delivery.

## Supporting Information

Table S1 **Sensitivity analyses: effect of study design on sample size with different screening test performance and treatment effects.**
(DOC)
