# Supplementary material for: Researching New Methods of Screening for Adverse Pregnancy Outcome: Lessons from Pre-eclampsia
Source: PLoS Med. 2012 Jul 31;9(7):e1001274. doi: 10.1371/journal.pmed.1001274 (PMC3409135; doi:10.1371/journal.pmed.1001274)
Supplement: Table S1 — Sensitivity analyses: effect of study design on sample size with different screening test performance and treatment effects. (DOC) [file pmed.1001274.s001.doc]

**Table S1. Sensitivity analyses: effect of study design on sample size with different screening test performance and treatment effects**

| Screening test & treatment | | | |  | Trial: randomise to screening | | |  | Trial: randomise high risk | | |  |
| --- | --- | --- | --- | --- | --- | --- | --- | --- | --- | --- | --- | --- |
| % screen positive | PPV  (%) | NPV  (%) | Treatment effect* |  | Incidence in screened | Incidence in unscreened | Sample size† |  | Incidence in intervention | Incidence in concealed | Sample  size† | Reduction in sample size (%) |
|  |  |  |  |  |  |  |  |  |  |  |  |  |
| 5 | 10 | 99.5 | 60 |  | 0.7% | 1% | 40,700 |  | 4% | 10% | 16,400 | 60 |
| 5 | 5 | 99.2 | 60 |  | 0.85% | 1% | 173,800 |  | 2% | 5% | 34,000 | 80 |
| 5 | 15 | 99.7 | 60 |  | 0.55% | 1% | 16,800 |  | 6% | 15% | 10,600 | 37 |
| 1 | 10 | 99.1 | 60 |  | 0.94% | 1% | 1,128,100 |  | 4% | 10% | 82,200 | 93 |
| 7.5 | 10 | 99.7 | 60 |  | 0.55% | 1% | 16,800 |  | 4% | 10% | 11,000 | 35 |
| 5 | 10 | 99.5 | 80 |  | 0.6% | 1% | 21,800 |  | 2% | 10% | 8,300 | 62 |
| 5 | 10 | 99.5 | 40 |  | 0.8% | 1% | 95,700 |  | 6% | 10% | 40,500 | 58 |

PPV denotes positive predictive value. NPV denotes negative predictive value. The background incidence in all the examples is 1%.

The top line presents the data illustrated in Figure 1.

*Percent reduction in incidence of primary outcome with treatment

† Sample sizes were based on a two sided test, with 90% power with alpha set at 0.05. In the case of randomisation to screening, the number is the total who would need to be consented prior to randomisation and is based on the predicted incidence in the screened group versus the incidence in the unscreened group. In the case of randomising high risk women to intervention or concealment of result, it is the total who would need to be screened and is based on the incidence in the high risk plus intervention versus high risk women where the result was concealed.
